# A cross-sectional study of the nutritional status of community-dwelling people with idiopathic Parkinson's disease

**DOI:** 10.1186/1471-2377-10-124

**Published:** 2010-12-30

**Authors:** Ahmed F Jaafar, William K Gray, Bob Porter, Elizabeth J Turnbull, Richard W Walker

**Affiliations:** 1 Department of Medicine, The Royal Victoria Infirmary, Newcastle upon Tyne, NE1 4LP, UK; 2 Department of Medicine, North Tyneside General Hospital, Rake Lane, North Shields, Tyne and Wear, NE29 8NH, UK; 3 The Medical School, University of Newcastle-upon-Tyne, NE1 7RU, UK

## Abstract

**Background:**

Parkinson's disease (PD) patients have an increased risk of under-nutrition, but we are unaware of any population based prevalence studies of under-nutrition in PD. The main objective of this study was to identify the prevalence, and nature, of under-nutrition in a representative population of people with PD.

**Methods:**

People diagnosed with idiopathic PD from within two PD prevalence study sites in North-East England were asked to participate in this study. Those who participated (n = 136) were assessed using a number of standard rating scales including Hoehn & Yahr stage and Unified Parkinson's Disease Rating Scale (UPDRS). Body mass index (BMI), mid-arm circumference (MAC), triceps skin fold thickness (TSF) and grip strength were recorded together with social and demographic information.

**Results:**

BMI < 20 identified over 15% of the study group to have under-nutrition. The Malnutritional Universal Screening Tool (MUST) scoring system identified 23.5% of participants at medium or high risk of malnutrition. Low BMI, indicating under-nutrition, was associated with greater age and disease duration, lower MAC, TSF, mid-arm muscle circumference (MAMC), reduced grip strength and a report of unintentional weight loss. Problems increased with increasing age and disease duration and were greater in females.

**Conclusions:**

Under-nutrition is a problem for around 15% of community dwelling people with PD. All PD patients should be screened for under-nutrition; the MUST score is a useful early screening tool.

## Background

Parkinson's disease (PD) is a progressive neurodegenerative disorder that has been shown to put individuals at increased risk of malnutrition and weight loss compared to age matched controls [1]. The nutritional problems associated with PD have been reviewed recently [2]. Previous studies have shown an increase in basal metabolic rate in PD patients, and this increase in energy expenditure has been implicated in the aetiology of weight loss in patients with PD [3-6]. Other possible causes of weight loss include, difficulty swallowing [7,8], inability to prepare food and handle it appropriately [9], gastrointestinal symptoms of the disease itself and drug side effects, such as dyskinesia [10,11].

Using the mini nutritional assessment as a screening tool, a recent 3 year longitudinal study of 61 PD patients from a referral centre found malnutrition risk increased from 22.9% to 34.3%. Greater disease duration was a significant predictor of risk from malnutrition [12]. The authors concluded that PD patients should be screened for nutritional status. Previous studies involving anthropometric measurement of PD patients have selected patients who were not representative of the wider PD community. Davies *et al *[5] studied 15 PD patients who had reported weight loss in the last 12 months and 15 controls. Although PD patients had significantly less body fat than controls, the authors noted the daily calorie intake of the PD patients to be significantly higher than the control subjects. We are unaware of any previous studies of the prevalence of nutritional problems in prevalent populations of people with PD.

The aim of this study was to assess the prevalence of under-nutrition in community-dwelling people with PD by use of standard anthropomorphic tests. Furthermore, we investigated whether levels of under-nutrition could be linked to demographic influences (age, sex, abode) and wider disease characteristics such as disease progression, disease duration, cognitive function, reported swallow problems and dyskinesia.

## Methods

In previously published prevalence studies in North-East England we identified 161 eligible cases of PD within an urban study population of 108,597 in the North Tyneside area and 106 cases within a predominantly rural study population of 59,613 in North Northumberland [13.14]. A nutritional investigation was carried out as part of these studies. Background data relating to age, gender, disease duration, functional limitations, motor and cognitive skills, disability, anxiety, depression, and quality of life were available for those who were eligible, and agreed to participate. For the nutritional component of the study, a proportion of the study population either declined to take part or was otherwise unable to participate.

All participants were assessed at home by a member of the study team. In North Tyneside this was a physician with an interest in movement disorders (B.P.) [13] and in North Northumberland this was either a physician with an interest in movement disorders or a PD nurse specialist [14]. Diagnosis of PD was confirmed using the UK PD Brain bank criteria [15]. Demographic information (age, sex, abode, duration of disease), Hoehn and Yahr stage (degree of disability and functional limitation), [16] and Unified Parkinson's Disease Rating Scale (UPDRS) score [17] was recorded from patient responses to verbal questioning, examination and assessment according to standard protocols. The researchers had all received training in the use of the UPDRS. The UPDRS is designed to measure and monitor progression in all aspects of PD and focuses on four key areas: 1) mentation, behaviour and mood, 2) coping with activities of daily living, 3) motor function and 4) changes due to complications of therapy. Cognition was assessed by administering the Mini-Mental State Examination (MMSE) [18].

Collection of nutritional data involved measurement of weight using calibrated portable scales and height using a steel tape measure. Patients were weighed without shoes, but with indoor clothing; no attempt was made to subtract the weight of the clothing. Patients' height was measured with their shoes removed. BMI was calculated using the formula weight (kg)/height^2 ^(m). A BMI of < 20 was taken as an indicator of under-nutrition, and a BMI of < 18.5 as severe under-nutrition [19,20] Mid-arm circumference (MAC), triceps skin fold thickness (TSF), and mid-arm muscle circumference (MAMC) were recorded. MAC was measured in cm at the mid-point between the olecranon and acromion process of the right arm. TSF was measured in mm at the same point using Harpenden calipers; 3 measurements were taken and then the mean value used for analysis. MAMC was calculated in cm using the formula MAC-(0.314 × TSF) [21]. Grip strength of the right upper limb was measured in kilograms using a dynamometer; the greatest value from 3 attempts was used in the analysis.

Patient reported problems in swallowing food, liquids or tablets (yes or no); alcohol intake (units of alcohol per week), whether the patient smoked or had ever smoked (yes or no) and unintentional weight loss of > 5% in the last 6 months (yes or no) were noted as these may contribute to under-nutrition. Other patient reported health problems were also noted. BMI and unintentional weight loss were used to calculate risk of malnutrition according to the Malnutrition Advisory Group's Mal-nutrition Universal Screening Tool (MUST) criteria [22].

This study was approved by the Joint Ethical Committee of Newcastle and North Tyneside and by the Northumberland Ethics Committee. Written informed consent was obtained from all subjects.

### Statistics

The data were quantitative in nature and collected at a nominal, ordinal and interval/ratio level. They were analyzed using standard statistical software, *SPSS-17 for windows *(SPSS, Chicago, IL, USA). With the exception of age and UPDRS score, all predictor variables were found to be non-normally distributed (Kolmogorov-Smirnov test) and so did not meet parametric assumptions. No obvious outlier or influential cases were identified. A cut off of p < 0.05 (two-sided test) for statistical significance was used throughout. Mann-Whitney U test was used to establish differences in age, disease duration, MMSE score, UPDRS score and Hoehn and Yahr stage for people for whom nutritional data were collected and for those whom this data were not collected; in the case of gender a Chi-squared test was used. Correlation between predictor variables were assessed using Spearman's method for ordinal and interval/ratio data, and using a point biserial method when one of the variables was dichotomous (gender, report of unintentional weight loss, dyskinesia present, BMI < 20).

## Results

Of the 161 prevalent cases of PD identified in North Tyneside, 135 (83.8%) consented to be assessed. Of these, 10 patients were excluded due to having a level of cognitive impairment that rendered anthropomorphic testing impossible; a further 2 patients were excluded due to physical disability. Due to the limited time and resources nutritional data were not collected on patients towards the end of the study period, but this should not have introduced any bias into the results as patients were seen in an arbitrary order. Nutritional data was collected on 82 of the remaining 123 patients (60.7%, 34 males, and 48 females). Of the 106 prevalent cases of PD identified in North Northumberland, 75 (70.8%) consented to be assessed, of whom 54 patients (72.0, 25 males, and 29 females) underwent nutritional assessment. There were no significant differences in age, sex, disease duration, disease stage (Hoehn and Yahr), cognitive function (MMSE) or disease progression (UPDRS score) between those who had nutritional data completed and those who did not (n = 53 for North Tyneside, n = 21 for North Northumberland), see Table 1. Therefore, we do not feel the fact that we could not assess all patients has biased our results in any significant way.

**Table 1:** Comparison of PD patients who underwent all nutritional and anthropometric tests with those who did not.

		Age	Disease duration	Hoehn and Yahr stage	MMSE score	UPDRS score	BMI	Grip strength (kg)
**North Tyneside**								
Data available (n = 82, 60.7%) 34 males, 48 females	Mean	74.59	5.61	2.49	26.80	30.96	24.88	18.72
	Std dev.	8.837	5.060	1.048	4.915	14.993	4.825	9.257
	Range	50-96	1-28	1-5	0-30	4-89	16.2-40.3	0-41
	95% CI	72.7-76.5	4.5-6.7	2.3-2.7	25.7-27.9	27.7-34.3	23.8-25.9	16.7-20.7
								
No data available (n = 53, 39.3%) 32 males, 21 females	Mean	75.83	5.79	2.60	23.76	33.59	-	-
	Std dev.	7.967	3.857	1.050	8.941	16.368	-	-
	Range	53-95	1-17	1-5	0-30	11-79	-	-
	95% CI	73.7-78.0	4.7-6.8	2.3-2.9	21.3-26.2	29.0-38.2	-	-
**North Northumberland**								
Data available (n = 54, 72.0%) 25 males, 29 females	Mean	75.33	4.52	2.60	25.09	36.96	26.49	16.40
	Std dev.	9.248	4.178	1.068	5.586	13.836	5.228	6.791
	Range	53-93	0-17	1-5	9-30	8-82	18.6-45.5	5.0-40.0
	95% CI	72.8-77.9	3.4-5.7	2.2-3.0	23.5-26.6	33.2-40.7	25.1-27.9	14.5-18.3
								
No data available (n = 21, 28.0%) 13 males, 8 females	Mean	74.25	5.81	2.50	26.84	38.62	-	-
	Std dev.	10.928	4.424	0.829	2.734	22.769	-	-
	Range	47-89	0-15	1-4	22-30	10-88	-	-
	95% CI	69.3-79.2	3.8-7.8	2.1-2.9	25.5-28.2	28.3-49.0	-	-
Difference between North Tyneside and North Northumberland*		U = 2036.5, z = -0.790, p = 0.432	U = 1787.5, z = -1.900, p = 0.057	U = 988.0, z = -0.570, p = 0.572	U = 1739.5, z = -1.980, p = 0.048	U = 1679.0, z = -2.381, p = 0.017	U = 1845.0, z = -1.641, p = 0.101	U = 1900.5, z = -1.058, p = 0.292

* For those with data available.

### Comparison of data for North Tyneside and North Northumberland

For those with nutritional data available, there was no significant difference in gender between those living in North Tyneside and those living in North Northumberland (χ^2 ^(1) = 0.310, p = 0.600). Likewise, as shown in Table 1 age, disease duration, Hoehn and Yahr stage, BMI and grip strength were not significantly different between patients from North Northumberland and North Tyneside. Mean MMSE score was 2 points lower and UPDRS score 6 points higher in North Northumberland. Given the broad similarity between the two groups, notably in terms of BMI and grip strength, data for North Tyneside and North Northumberland patients were combined for further analysis.

### Combined data set

There was a negative correlation between BMI score and age (r = -0.273, p = 0.001), disease duration (r = -0.234, p = 0.006) and a report of unintentional weight loss by the patient (r = -0.378, p < 0.001). There was a positive correlation with grip strength (r = 0.210, p = 0.013) MAC (r = 0.664, p < 0.001) TSF (r = 0.349, p < 0.001) and MAMC (r = 0.534, p < 0.001). BMI score was not associated with smoking, alcohol consumption, dysphagia, dyskinesia, living in institutional care, UPDRS score or MMSE score.

Those who did have under-nutrition (BMI < 20, n = 21) were compared to those who did not (n = 115); results are given in Table 2; for anthropomorphic tests, results are split into males and females. Seven participants (5.15%) had a BMI below 18.5, indicating severe under-nutrition, of whom 5 were female. Using the MUST scoring system 104 patients (56 females) were at low risk of malnutrition, 21 patients (11 females) were at medium risk of malnutrition and 11 (10 female) patients were at high risk of malnutrition. The odds of a female being at high risk of malnutrition compared to a male are 11.5 (95% CI 1.43 to 93.0).

**Table 2:** Comparison of those with BMI below 20 with those with BMI above 20.

	BMI < 20 (n = 21, 15.4%)		BMI ≥ 20 (n = 115, 84.6%)		
	Median	Range	Median	Range	Significance
**All participants**	6 male, 15 female		53 male, 62 female		χ^2 ^(1) = 2.218, p = 0.137
Age	81	70-92	75	50-96	U = 760.0, z = -2.696, p = 0.006**
Disease duration	6.0	1-13	3.5	0-28	U = 869.5, z = -2.039, p = 0.041*
Hoehn and Yahr	3.0	1-5	2.5	1-5	U = 712.5, z = -1.585, p = 0.114
MMSE	28	10-30	28	0-30	U = 1115.5, z = -0.502, p = 0.619
UPDRS	37	5-60	32	4-89	U = 939.0, z = -1.618, p = 0.106
					
**Males**	n = 6		n = 53		
Grip strength (kg)	16.0	10-29	21.0	6-41	U = 89.0, z = -1.206, p = 0.239
MAC (cm)	23.0	19-36	29.0	22-36	U = 72.5, z = -1.543, p = 0.129
TSF (mm)	7	5-11	11	4-25	U = 61.0, z = -1.920, p = 0.055
MAMC (cm)	21.0	16.5-33.8	25.2	18.7-32.2	U = 74.0, z = -1.447, p = 0.155
Unintentional weight loss	1 yes, 5 no		5 yes, 48 no		χ^2 ^(1) = 0.309, p = 1.000
					
**Females**	n = 15		n = 62		
Grip strength (kg)	11.0	5-19	15.0	0-28	U = 301.0, z = -2.074, p = 0.040*
MAC (cm)	22.0	17-26	28.0	16-38	U = 94.0, z = -4.670, p < 0.001**
TSF (mm)	10	8.0-14.0	14	6-37	U = 221.5, z = -3.044, p = 0.002**
MAMC (cm)	18.7	14.3-23.3	22.2	12.2-33.8	U = 158.5, z = -3.775, p < 0.001**
Unintentional weight loss	8 yes, 7 no		6 yes, 56 no		χ^2 ^(1) = 15.474, p = 0.001**

* Significant at the p < 0.05 level

** Significant at the p < 0.01 level

Table 3 compares our anthropomorphic data with data from two studies of the general elderly. One study is set in an industrial area of South Wales [23] in the early 1970 s and the other is from Edinburgh [24] in the mid-1990 s.

**Table 3:** Comparison of our cohort with reference data from other studies

	North Tyneside and North Northumberland PD patients (median)	Edinburgh 75-79 years (mean)^23^	South Wales 75-79 years (median)^24^
Males			
BMI (kg/m^2^)	24.8	26.9	23.9
MAC (cm)	29.0	29.7	24.9
TSF (mm)	10.5	11.4	7.0
MAMC (cm)	24.8	26.1	22.1
			
Females			
BMI (kg/m^2^)	25.0	26.2	26.1
MAC (cm)	27.0	29.9	24.9
TSF (mm)	13.0	17.1	14.6
MAMC (cm)	21.8	24.5	20.0

## Discussion

This is the first published study, as far as we are aware, of nutritional problems in prevalent PD populations. Whilst the study is limited by a lack of data collection in a proportion of individuals it is reassuring that the disease characteristics of those in whom data was collected is not significantly different from those in whom data was not collected, as evident from the overlap of 95% confidence intervals in all cases.

Although there is some debate as to the ideal BMI range for older people, a cut-off of < 20 for under-nutrition is widely recognised by most dieticians [25]. Moreover, a BMI of < 20 has been suggested as a significant predictor of short-term mortality in older adults (mean age 73.5 years) [19]. Using BMI as a marker, under-nutrition was a problem in over 15% of community-dwelling people with PD. This compares with a report of only 3.6% under-nutrition (BMI < 20) in an Italian study of nutrition in 3356 randomly selected elderly people (age range 65-84) [26]. Using the MUST criteria 23.5% of our cohort was at medium or high risk of malnutrition (8.1% at high risk). Considering females only these figures rise to 27.3% at medium to high risk, with 13.0% at high risk. Use of both BMI and a report of unintentional weight loss in the scoring system means that patients who still have an acceptable BMI, but are likely to become undernourished in the future if they continue to lose weight, are identified earlier than if BMI alone is used as a screening tool. This is likely to be particularly important in PD patients where weight loss may be due complex disease related factors, and therefore easier to control through dietary intervention if recognised at an early stage. Interestingly, unintentional weight loss was a stronger predictor of under-nutrition than patient reported swallowing problems.

We have not attempted to categorise patients according to degrees of malnourishment based on our anthropometric findings, since this would require the use of reference data from comparable normal populations. Data from 3 industrial areas of South Wales in the early 1970 s, although extensive, is likely to be out of date due to improving standards of health amongst the general elderly population over the last 30 years [23]. The average BMI of both males and females in our cohort was lower than that seen more recently in a study of elderly people in Edinburgh [24]. MAC, TSF and MAMC averages across both genders were generally higher than those recorded in South Wales, but lower than those recorded in Edinburgh. Although it is difficult to draw firm conclusions from these observations it may be that the Edinburgh cohort, being more recent, are a better match for our cohort and that our cohort are undernourished when compared to the general elderly population of a similar age.

Greater age and longer disease duration were significantly associated with under-nutrition in our cohort. Surprisingly, UPDRS score and Hoehn and Yahr stage, both markers of disease progression, were not significantly associated with under-nutrition. The reason for this is not clear, although comparison with previous research suggests there may be a link. The cohort of Davies *et al *[5] were of later Hoehn and Yahr stage than our cohort and had lower BMI and TSF and higher MAMC, indicating that late stage PD patients may have a greater degree of under-nutrition, with fat, rather than muscle, loss accounting for the change. Markus *et al *[27] studied 95 PD patients from a neurological unit. Despite the fact that the mean age of the cohort was only 61.7 years, the average Hoehn and Yahr stage was 3.0 and the average disease duration was 9.1 years, indicating the patients to be amongst the most severely affected by the disease. Once again, BMI and TSF were lower and MAC higher than for our cohort. Our study of patients at an apparently earlier disease stage tends to support the view that those earlier in the disease cycle have fewer problems of under-nutrition, as noted by others [12]. Further research in this area is required.

Measurements of grip strength, MAC and TSF were highly correlated with having a BMI below 20 in females, but not males. Consideration of median and range of the data suggests that this lack of statistical significance is due to the relatively low number of males with BMI below 20, rather than any inherent anthropomorphic reason. Anthropomorphic measurements may offer an alternative method of assessing PD patients for nutritional risk, where height and weight are difficult to assess. Indeed, anthropomorphic measurements have been shown to be significant predictors of mortality in older people [28].

Whilst these are prevalence studies, we accept that the prevalence estimate is only a minimum estimate and that not all cases of PD will necessarily have been identified, and that not all of the prevalent population participated in the nutritional study. We suspect the real prevalence of under-nutrition to be even higher taking into account that those with cognitive impairment and severe disability were more likely to be excluded.

## Conclusions

Under-nutrition is a problem for 15% of a prevalent population of community-dwelling people with PD in North-East England and almost a quarter (23.5%) are at medium or high risk of malnutrition. Women are at greater risk from severe under-nutrition than men. However, the prevalence of under-nutrition increases with age and disease duration. Clinicians should screen for, and monitor, nutrition in all PD patients and should consider use of the MUST screening tool as an early indicator of nutritional problems.

## Competing interests

The authors declare that they have no competing interests.

## Authors' contributions

This study was conceived, organised and executed by Prof. Richard Walker and Dr Bob Porter. Primary data collection was by Dr Bob Porter for North Tyneside and by Prof. Richard Walker and research nurses Catherine Jones and Sheila Durkin for North Northumberland. Statistical analysis and writing of the first draft of the paper was done by Dr William Gray, Dr Ahmed Jaafar and Miss Elizabeth J. Turnbull. All listed authors were involved in the preparation, review and critique of the final manuscript.

## Pre-publication history

The pre-publication history for this paper can be accessed here:

http://www.biomedcentral.com/1471-2377/10/124/prepub
